# The Effects of Cranberry Polyphenol Extract (CPE) Supplementation on Astringency and Flavor Perception as a Function of PROP Taster Status and Other Individual Factors

**DOI:** 10.3390/ijerph191911995

**Published:** 2022-09-22

**Authors:** Neeta Y. Yousaf, Beverly J. Tepper

**Affiliations:** Department of Food Science & Center for Sensory Sciences & Innovation, Rutgers University, New Brunswick, NJ 08901, USA

**Keywords:** astringency, PROP phenotype, cranberry, polyphenols, machine learning, taste

## Abstract

This study investigated whether PROP (6-n-propylthiouracil) taster status and other individual factors (gender, ethnicity, BMI, and age) are markers of variation in perceptions of astringency and other flavor attributes. Participants (*n* = 125) evaluated cranberry juice cocktail samples (CJC) supplemented with cranberry-derived polyphenol extract (CPE, added at 0, 0.3, 0.5 and 0.75 g/L), as well as control samples, unsweetened cranberry juice (CJ) and an aqueous solution of 0.75 g/L CPE. Subjects evaluated samples for key sensory attributes and overall liking using a 15 cm line scale. The data were analyzed using ANCOVA and machine learning tools (regression trees and random forest modeling) to examine if the latter approach would extract more meaningful insights about the roles of personal factors in sensory perceptions of cranberry-derived stimuli. ANCOVA revealed robust stimulus effects, but no effect of PROP taster status on astringency perception was observed. Several effects of PROP×gender, ethnicity, and other factors were observed on other sensory attributes and liking. ANCOVA and machine learning tools yielded similar findings, but regression trees provided a more visualized framework. These data show that PROP taster status has a limited role in astringency perception in complex samples and that other personal factors deserve greater focus in future research on astringency perception.

## 1. Introduction

Astringency is a well-known tactile sensation marked by drying, puckering, and roughing of the oral surfaces [1] upon the consumption of polyphenol-rich foods. Although astringency is an expected and often desirable trait in certain foods, it can also be viewed as an unpleasant sensation. Indeed, numerous studies show that astringency can be a negative driver of acceptance of many foods and beverages including but not limited to tea [2], dark-colored fruits [3] and juices [4,5,6], whey beverages [7], cheese [8], nuts [9], chocolate [10], and wine [11]. Astringency has also been associated with consumer perceptions of lower quality [4,12].

Polyphenol-rich foods provide substantial health benefits, ranging from anti-inflammatory properties; protection from cardiovascular diseases, obesity, and metabolic syndrome; to contributing to a healthy oral and gut microbiota [13,14,15,16,17]. Public health policies in the U.S. and elsewhere strongly encourage the consumption of fruits and vegetables [18] that are polyphenol-rich, but their bitterness and astringency can serve as barriers to their acceptance especially among individuals who are highly sensitive to these attributes. Thus, obtaining a better understanding of individual differences in astringency perception may lead to better strategies for designing and marketing products that will appeal to a broad range of consumers. Since food preferences are also shaped by differences in familiarity, diet, gender, and ethnicity, as well as personal traits such as food neophobia, nutrition attitudes, sensitivity to disgust, and sensitivity to reward and punishment among others [19,20,21,22,23,24], it is also important to assess the role of these diverse factors in studies. One of the factors that has been investigated in this area of research is the role of the *TAS2R38* gene.

Variation in *TAS2R38*, the gene that controls the ability to perceive 6-n-propylthiouracil (PROP) is a common marker for individual differences in bitterness perception and other oral sensations such as alcohol burn, pungency, and oral–tactile perception as well as astringency. PROP taster status is the phenotypical manifestation of polymorphisms in the *TAS2R38* gene, which codes for the bitter taste receptor TAS2R38. Individuals with homozygous dominant alleles for this gene (PAV/PAV) experience greater bitterness from common foods and are called super-tasters (STs) [25,26]. Individuals who are recessive for this gene (AVI/AVI, phenotypic non-tasters, NTs) experience little PROP bitterness in comparison to STs; heterozygotes (medium tasters, MTs) perceive moderate bitterness from PROP. Generally speaking, PROP STs perceive greater intensities of the above mentioned oral sensations in comparison to NTs which reduces their preferences for, and selection of foods with these sensory qualities [27].

Studies specifically investigating the influence of PROP status on the acceptance and consumption of astringent foods have produced mixed results. Pickering (2004; 2006) reported that PROP STs experienced greater astringency from red wines in comparison to NTs [28,29]. Contrary to what was expected, Laaksonen et al. (2013) reported that PAV homozygotes (phenotypical PROP STs) gave lower astringency ratings to berry juice fractions supplemented with polyphenols [30] than AVI homozygotes (phenotypic PROP NTs). Nevertheless, the PAV homozygotes disliked the samples the most. More recently, Melis and colleagues (2017) [31] showed that PROP taster status played a role in the perception and liking of cranberry juice cocktail modified to enhance astringency, but only when examined separately by gender. Specifically, they reported that PROP ST males perceived greater bitterness and astringency from cranberry juice cocktail supplemented with tannic acid and gave lower liking ratings to it than did non-taster males [31]. Other researchers have been unable to find robust evidence of such relationships [32,33,34,35].

There are numerous reasons for the discrepancies across studies. First, differences may lie in the type of astringent stimulus used. In the early studies, alum, tannic acid, or grape seed tannin were used as model stimuli [33,36,37] which are not consumed on their own in typical diets. Since the intensity and quality of astringency is highly dependent on the type of astringent stimulus [36], extrapolating the findings from laboratory stimuli to real-world complex foods, such as fruit juices may not be valid. Second, study populations tested in these studies have varied widely. Many studies utilized homogeneous populations (typically majority White Caucasian populations from North America and Western Europe) [35,37]; some examined more ethnically-diverse consumer groups [38,39], and others did not report on the ethnic mix of their participants [8,30,40,41]. The lack of ethnic diversity and balance in taste studies is not unique to studies on PROP status and astringency. When Burgess et al. (2018) examined the role of *CD36* polymorphisms on fatty acid taste sensitivity they tested a well-described cohort of Caucasian and East Asian participants [42]. By doing so, they were able to demonstrate different gene effects in the two ethnic groups that were unobservable when all subjects were examined as a single group. Thus, the investigation of astringency with more weight placed on personal characteristics using ecologically representative food models deserves more attention. Yet another difference may lie in the data treatment or type of statistical test used. Studying more than a few individual factors of variation with traditional statistical testing (e.g., analysis of variance) often yields complex outputs, with the presence of two- or three-way interactions, challenging a meaningful interpretation of the data. Recent work [23,42,43] has used machine learning tools, specifically regression trees, to distill complex sensory datasets into visualized frameworks. Thus, there is an opportunity to use machine learning to uncover the complex relationships between astringency perception and individual factors of variation.

The first objective of the present study was to investigate if PROP taster status and other individual factors such as age, gender, ethnicity, and BMI are markers of variation in perception of astringency and other flavor attributes of cranberry juice. Similar to our recent work on polyphenols and salivary protein release [39], we used cranberry juice and cranberry juice cocktail that we supplemented with cranberry-derived polyphenols as an ecologically relevant model stimulus. We hypothesized that PROP STs would perceive greater bitterness, astringency, and intensity of other flavor attributes in comparison to NTs, and that this would negatively affect their liking of cranberry juice.

The second objective of the study was to utilize a machine-learning based modeling approach to study these research questions. Traditional analysis of covariance (ANCOVA) was used alongside machine learning models (regression trees and random forest modeling) to determine if the latter methods extracted more meaningful information from the data, particularly with respect to parsing out the role of individual variability.

## 2. Materials and Methods

### 2.1. Subject Recruitment

A total of 125 healthy adults (62 males and 63 females) age 18–45 years were recruited from the Rutgers University community through an email distribution list. Subjects were screened for PROP taste sensitivity (see Section 2.2 below), and only PROP NTs and STs were admitted into the study into groups balanced for gender. Subjects were also screened for general suitability (e.g., demographics, health information), familiarity with cranberry juice and products, and consumption within the last 2 years. Demographic information collected in this study included gender, age, ethnicity, country of birth, and length of time living in the U.S. Only subjects who had lived in the U.S. for at least 1 year were admitted into the study. 

Exclusion criteria included major metabolic diseases (diabetes, kidney disease, etc.), pregnancy, lactation, food allergies, and the use of medications that interfere with taste or smell functions (e.g., steroids, antihistamines, or anti-depressants). Participants who were determined to be PROP MTs were also excluded.

The study was approved by the Rutgers University Arts and Sciences Institutional Review Board (Approval#13-309M). All subjects provided informed consent and were compensated monetarily for their participation.

### 2.2. PROP Taster Status

The participants were screened and classified according to PROP taster status via the paper disk method [40], which has previously been tested for validity and reliability [31,44] and strongly correlates with tongue electrophysiological recordings [41,45].

Briefly, subjects place a filter paper disk impregnated with 1.0 mol/L NaCl on the tip of the tongue until it is thoroughly wet. They rate the taste intensity of the disk using the labeled magnitude scale (LMS), a 100-mm scale anchored with the phrases “barely detectable” to “strongest imaginable”. This procedure is repeated with a second paper disk impregnated with 50 mmol/L PROP (6-n-propyl–2-thiouracil, P3755, Sigma-Aldrich, St. Louis, MO, USA). Subjects are instructed to rinse with spring water at room temperature before and in between tasting each paper disk. Subjects are categorized as NTs if they rate the PROP disk <15 mm on the LMS; they are categorized as STs if they rate the PROP disk >67 on the LMS. All others are classified as MTs. NaCl ratings do not vary with PROP status in this method. Therefore, NaCl ratings are used as a reference standard to clarify the taster status of subjects who give borderline ratings to PROP. This strategy is based on the rationale that NTs give higher ratings to NaCl than to PROP, MTs give equivalent ratings to both stimuli and STs give higher ratings to PROP than NaCl [40].

### 2.3. Test Stimuli

This study used cranberry juice with different concentrations of cranberry polyphenol extract (CPE) (to modulate astringency) as a test stimulus.

There were a total of six samples in this study: CJ and CPE (aqueous solution) as controls and CJC supplemented with zero, Low, Med or High CPE as the test samples. Table 1 shows the details for each of these samples.

Cranberry juice (CJ) was made from frozen cranberries donated by Ocean Spray (Chatsworth, NJ, USA). The cranberries were frozen at −20 °C until use. Cranberry juice was made in small batches to maintain freshness using a standard recipe. Briefly, 300 g of berries was defrosted, washed, and cooked on a stovetop with 648 mL of spring water. The mixture was filtered through cheesecloth and allowed to cool. Cranberry juice cocktail (CJC) was made from 600 mL of warm CJ to which was added 52.5 g of sucrose. The final concentration of sucrose in the CJC was 87.5 g/L (8.75% *w*/*v*). Batches of CJ and CJC were prepared as needed, the day before subject testing, and refrigerated at 4 °C until 30 min before use.

The cranberry-derived polyphenol extract solution (CPE) was made using a carrier-free powdered extract (Ocean Spray, Middleborough, MA, USA). Powdered CPE was added to pre-warmed spring water at a concentration of 0.75 g/L, while stirring on a hot plate to facilitate dissolution. This solution was prepared as needed, the day before subject testing, and refrigerated at 4 °C until 30 min before use.

Cranberry juice cocktail was supplemented with CPE in three concentrations: at 0.3 g/L (Low), 0.5 g/L (Med) and 0.75 g/L (High). These concentrations were selected after extensive benchtop screening for palatability. The lowest concentration (i.e., 0.3 g/L) could be discriminated from the standard CJC in pilot testing. Beyond the highest concentration of CPE (i.e., 0.75 g/L), the CJC was no longer palatable. For these samples, CPE powder in the respective concentrations was mixed directly into freshly prepared CJC. These samples were prepared and handled in the same manner as above.

All samples were served at room temperature. Order of presentation was randomized and there was a forced 5 min break in between sample evaluations.

### 2.4. Taste Ratings

Intensity ratings for key sensory attributes of the cranberry-based beverages were collected using standard, 15 cm line scales anchored with the phrases “very weak” to “very strong.” Attributes of interest included sweetness, bitterness, sourness, astringency, thickness, cranberry flavor, and overall flavor. Overall liking of the samples was collected using a similar scale anchored with “dislike extremely” and “like extremely.” Sensory ratings were collected electronically using RedJade data collection software, where the ballot is presented on a computer screen and subjects make their assessments electronically.

### 2.5. Experimental Procedures

Subjects were instructed to refrain from consuming astringent foods for approximately 8 h prior to each session. A list of such foods was provided to them. They were also prohibited from eating, drinking (except plain water), chewing gum, using breath mints, mouthwash or brushing their teeth for 2 h prior to the test sessions.

Subjects participated in three test sessions scheduled at the same time on three different days. All testing took place in individual testing booths. The entire testing sequence took approximately 3 weeks to complete.

**Figure 1:**
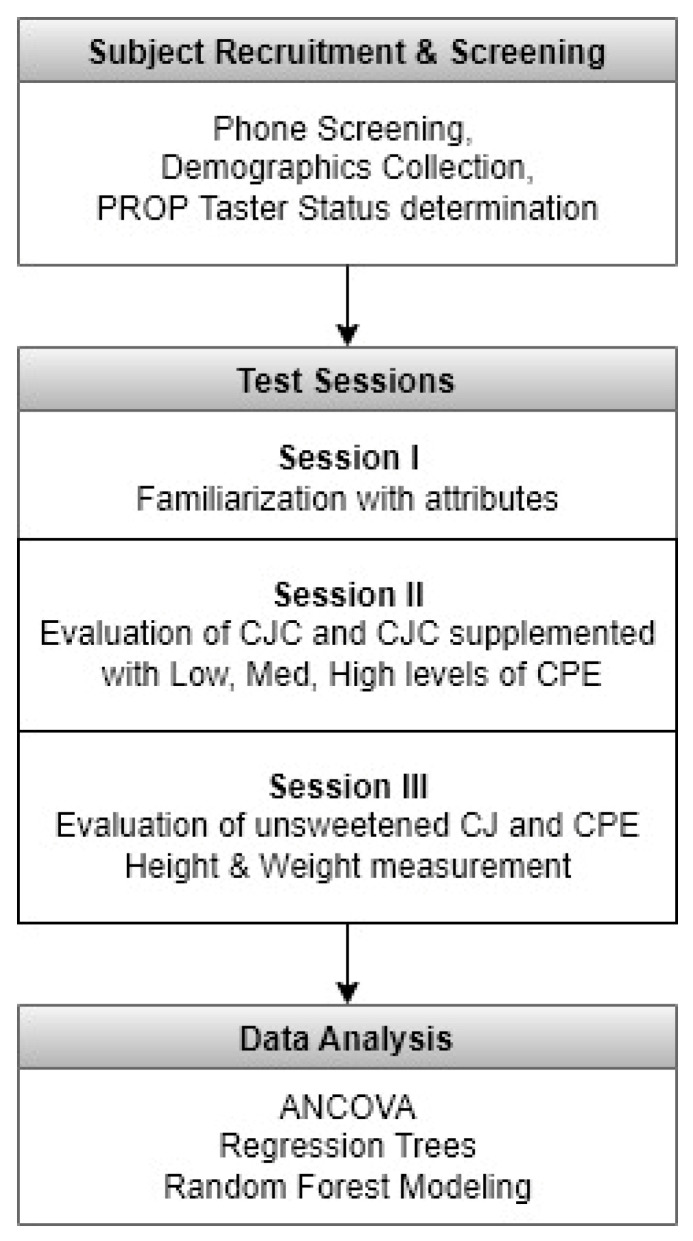
Experimental Procedure.

Figure 1 shows the experimental procedure.

#### 2.5.1. Session I: Familiarization Task

During session 1, subjects were presented with prototypical reference solutions for sourness (1.9 g/L citric acid), bitterness (0.6 g/L caffeine) and astringency (1.38 g/L alum) to familiarize themselves with these attributes. Subjects then participated in a matching exercise where they were asked to identify the aforementioned reference solutions with the correct sensory attribute. If a subject answered incorrectly, they were provided additional opportunities to practice. Finally, subjects then practiced evaluating these attributes by rating the intensity of sourness, bitterness, and astringency of a plain cranberry juice cocktail (CJC) sample.

#### 2.5.2. Session II: Sample Evaluations

Subjects evaluated the various CJC samples with added CPE (see Table 1 above) with 5 min breaks in between sample evaluations.

#### 2.5.3. Session III: Sample Evaluations & Height and Weight Measurements

Subjects evaluated unsweetened cranberry juice (CJ) as well as CPE in water using the same procedure as in session 2. Height and weight were also measured for body mass index (BMI) calculations (kg/m^2^).

### 2.6. Data Analysis

XLSTAT Software (Addinsoft, New York, NY, USA) was used to perform all statistical analyses. Normality testing was conducted using the Shapiro–Wilk test, while homogeneity of variances was tested using Levene’s test.

#### 2.6.1. ANCOVA

An ANCOVA model was applied to determine effects of the independent variables on the intensity of the sensory attributes and overall liking ratings. Independent variables were a combination of grouping factors and covariates. PROP taster status, gender, ethnicity, and sample type were used as the main factors, while age and BMI were the covariates. Post hoc testing was conducted for sample type using Duncan’s test (*p* < 0.05).

#### 2.6.2. Regression Trees

To explore the complex and multifactorial nature of the dataset, regression trees were used as a secondary approach to examine the data. CHAID regression trees were computed, in which an F test is conducted for each explanatory variable. If a significant difference is found (*p* < 0.05), a node is created. Each node was set to 5% difference. Next, validation was performed for each tree, where a subset of data was randomly selected (*n* = 100) and the explanatory power of the tree was tested. The fits of the trees were compared using the mean square error (MSE), and the models were optimized until the MSE was minimized as much as possible.

The trees were computed separately for each of the attributes using the same factors as in the ANCOVA model above. Additionally, age was converted to a discrete factor with two levels: Under 21 years or Over 21 years of age. Similarly, BMI was converted to a discrete variable with two categories: Normal (≤25 kg/m^2^) or Overweight (>25 kg/m^2^).

#### 2.6.3. Random Forest Modeling

It is well known that regression trees are prone to overfitting a dataset so that even robust significant effects may be overestimated and not generalizable to a larger dataset from a representative population [46]. In comparison, random forest modeling builds many regression trees, each using a random subset of the data. In this manner, the data is sampled repeatedly. The final model presents averaged data across the trees, thus minimizing overfitting of the model.

A random forest model was used here as a qualitative check on the validity of the regression trees using the same independent variables as described. Specifically, results were averaged across 1000 trees and feature importance scores were generated i.e., a score was assigned to each factor in how important it was in explaining variance in sensory attribute ratings and overall liking scores. A heatmap was then computed showing the rank order of the relative importance of each factor in explaining variance in a given attribute.

## 3. Results

Table 2 shows subject characteristics.

The subject pool was mostly Caucasians (*n* = 77), and the rest were Asians (*n* = 48). Mean participant age and BMI were 21.8 ± 0.4 years and 24.9 ± 0.4 kg/m^2^, respectively. Taster by gender subgroups were approximately balanced.

### 3.1. ANCOVA Results

ANCOVA results revealed several significant effects of individual factors of variation on the sensory ratings (Table 3).

**Figure 2:**
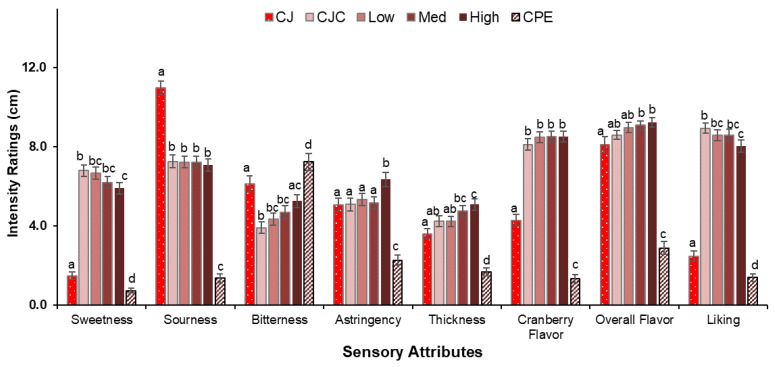
Effect of sample type on attribute intensity ratings (Mean ± SEM) of the CJ, CJC, Low, Med, High and CPE samples. Different superscripts (a, b, etc.) denote statistically significant differences within an attribute based on post-hoc analyses (*p* < 0.05).

The most prominent effect was sample type, which significantly affected ratings of all sensory attributes as well as liking (Figure 2).

The two controls, CJ and CPE were generally perceived to be distinct from the four CJC samples, which were clustered more closely. CPE received the lowest intensity ratings for all sensory attributes except bitterness and was liked the least. Among the four CJC samples, CJC was the sweetest, while High was rated as having the most bitterness, thickness, and overall flavor and was liked the least. Low and Med samples were given intermediate ratings.

Several other significant effects were also revealed by the ANCOVA (Table 3). An overall main effect of ethnicity was found for three attributes: bitterness (*p* < 0.0001), astringency (*p* = 0.002), and liking (*p* = 0.000). Overall, Caucasian subjects gave higher-intensity ratings to both bitterness and astringency in comparison with Asians, who liked the samples less than Caucasian subjects did.

There were also two significant interactions. First, a gender×sample (*p* = 0.015) interaction affected sourness ratings. This was mainly due to male subjects giving higher sourness ratings to CJ in comparison with females (11.8 ± 0.4 vs. 10.3 ± 0.5 respectively). Second, a taster×gender interaction was observed for sourness (*p* = 0.001) and cranberry flavor (*p* = 0.000) attributes. Specifically, female STs gave higher ratings to these attributes than female NTs. No differences were observed in male subjects with respect to PROP groups.

### 3.2. Regression Trees

**Figure 3:**
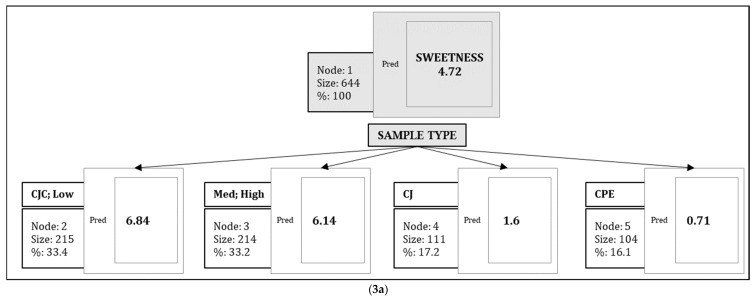

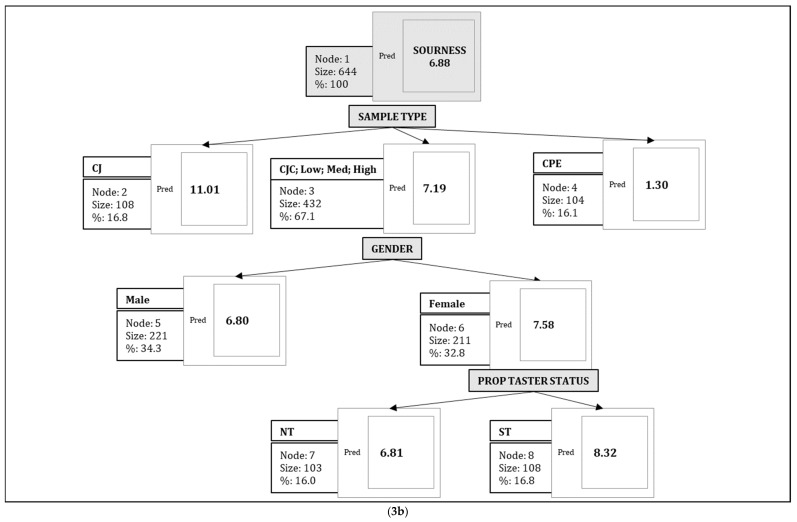

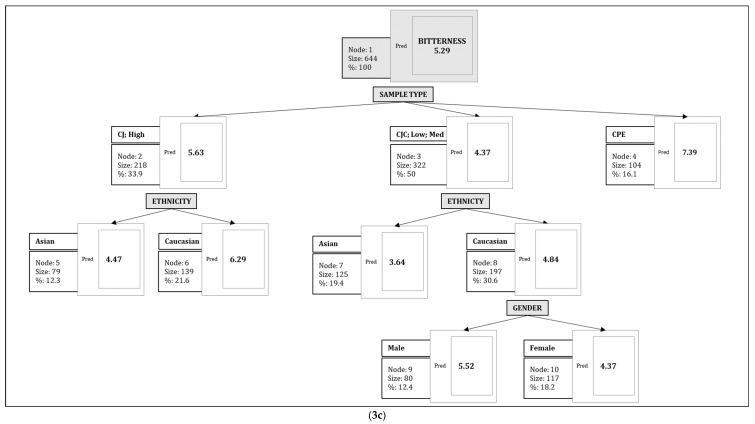

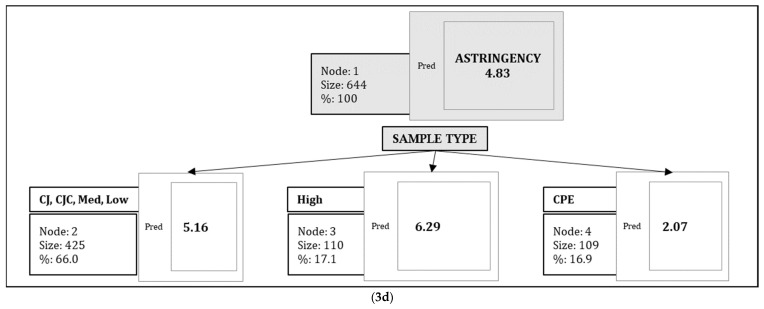

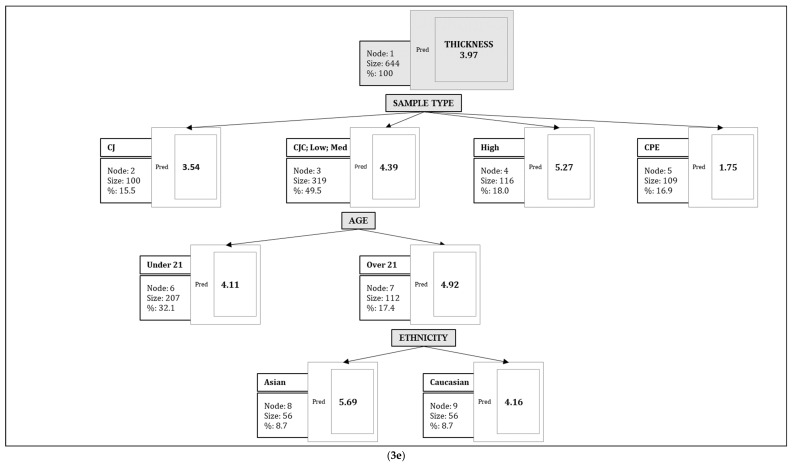

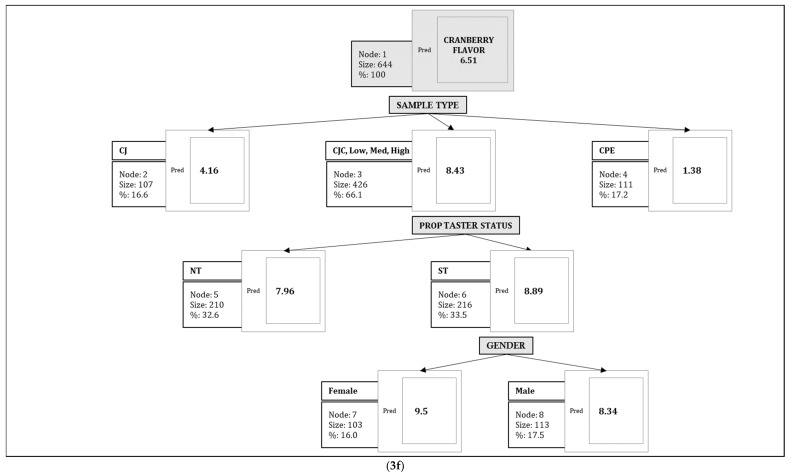

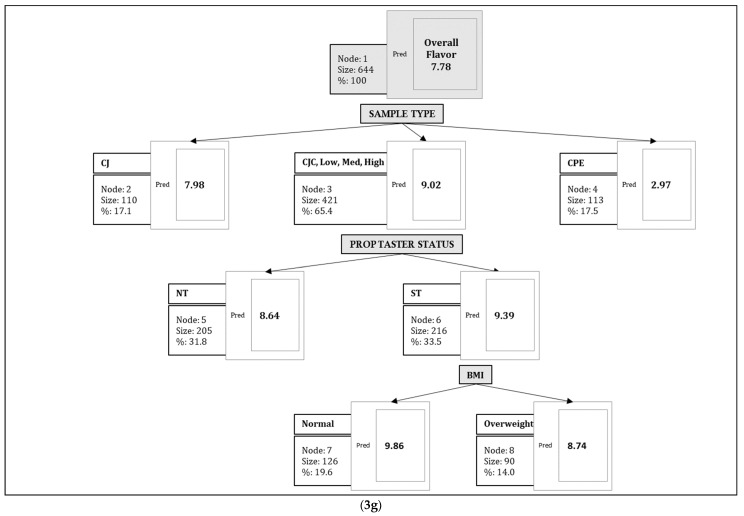

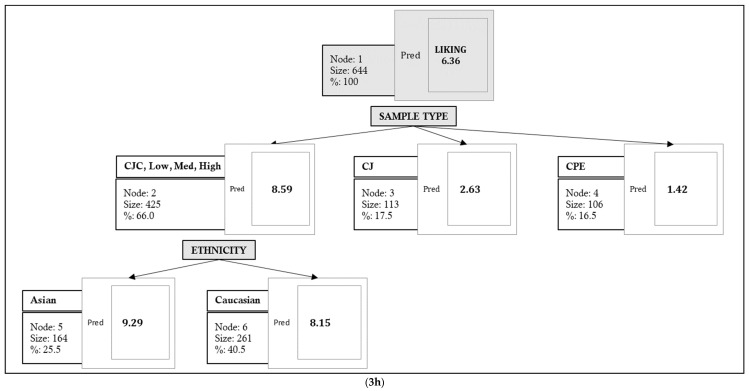
(**3a**): Regression tree for sweetness intensity ratings. Node refers to a split based on a significant test (*p* < 0.05). Size refers to number of subjects in a given split while % refers to the corresponding percentage of the total subjects in a given split. ‘Pred’ shows predicted values of intensity ratings on the 15-cm line scale. (**3b**): Regression tree for sourness intensity ratings. (**3c**): Regression tree for bitterness intensity ratings. (**3d**): Regression tree for astringency intensity ratings. (**3e**): Regression tree for thickness intensity ratings. (**3f**): Regression tree for cranberry flavor intensity ratings. (**3g**): Regression tree for overall flavor intensity ratings. (**3h**): Regression tree for overall liking ratings.

Figure 3 show regression trees computed for each of the attributes.

For all the trees, sample type was always the first node in the tree, which indicated that the intensity for each attribute including liking was most dependent on sample type. Within the ‘Sample’ node, the CPE sample was always a separate branch, and the grouping of other samples varied with each specific sensory attribute. The details of these groupings are described below.

For sweetness (Figure 3a) and astringency (Figure 3d), the trees terminated at sample type, and no other independent variables were found to have significant explanatory power.

For the remaining attributes, explanatory variables ranked differently in predictive power.

For sourness (Figure 3b), eight terminal nodes were generated. The first variable was sample type, which separated into three nodes: CJ, CPE, and the CJC samples (grouped together). A further partition was observed for the CJC samples (node 3) based on the variable gender. Females gave higher sourness ratings than males (*p* = 0.016). Within female subjects (node 6), PROP taster status was a significant explanatory variable of sourness ratings, where female STs rated sourness higher than did female NTs (*p* = 0.001).

For bitterness (Figure 3c), after sample type, ethnicity accounted for the most variance. First, three nodes emerged from Sample Type: CJ and High (grouped together), CJC/Low/Med (grouped together) and CPE alone. Within the first two groupings (nodes 2 and 3), ethnicity was a significant factor, where Caucasian subjects rated bitterness higher in comparison to Asian subjects (*p* = 0.002−0.001). Within the CJC/Low/Med samples (node 3), there was a further split based on Gender, wherein male Caucasian subjects perceived greater bitterness from these samples than female Caucasian subjects (*p* = 0.021).

For thickness (Figure 3e), sample type had the most explanatory power. Within the CJC samples (node 3), age was a significant predictor, where subjects Over 21 years of age rated thickness as higher than those Under 21 years of age (*p* = 0.023). Additionally, within the node Over 21 years of age (node 7), ethnicity had a significant effect, wherein Asian subjects rated thickness higher than Caucasian subjects (*p* = 0.008).

For cranberry flavor (Figure 3f), the first significant factor was sample type, which split into three nodes: CJ, CJC/Low/Med/High (grouped together), and CPE. Within the CJC sample types (node 3), there was a further split on PROP taster status, where STs gave higher ratings to cranberry flavor than NTs (*p* = 0.002). Within STs (node 6), there was a split based on gender, whereby female STs gave higher ratings to cranberry flavor than male STs (*p* = 0.004).

For overall flavor (Figure 3g), the first significant factor was sample type, which split into three nodes: CJ, CJC/Low/Med/High (grouped together) and CPE. Within the CJC sample types (node 3), there was a further split on PROP taster status, where STs gave higher ratings to overall flavor than NTs (*p* = 0.004). There was a further split that emerged from the ST subjects (node 6), where BMI was a significant predictor. Subjects with a BMI in the normal range gave higher ratings to overall flavor than those who were overweight (*p* = 0.002).

Finally, for overall liking (Figure 3h), the first split was on sample type, which was partitioned into three nodes: CJ, CPE, and CJC/Low/Med/High (grouped together). Within CJC samples node (node 2), there was a split on ethnicity, with Asian subjects, giving significantly higher liking ratings to these samples than Caucasian subjects (*p* = 0.000).

### 3.3. Random Forest Modeling

Random forest modeling was conducted for each of the sensory attributes as well as liking. Results are summarized in a heatmap using feature importance based on mean square error values (Table 4).

Across all the attributes, sample type was the single most important factor that influenced attribute intensity and overall liking ratings. However, after sample type, each feature ranked differently in its explanatory power for each sensory attribute. Notably, all importance scores for other factors were much lower than the sample type importance score.

For sweetness, PROP taster status and BMI were the most important after sample type. For sourness and cranberry flavor, rankings were similar: after sample type, PROP taster status and gender were the most important factors. For both bitterness and astringency, ethnicity was the most important factor after sample type and had a higher importance score than PROP taster status. Age was the most important variable for thickness after sample type. For overall flavor, BMI was most important after sample type, followed by PROP taster status and gender. Finally, like bitterness and astringency, ethnicity had the most impact on overall liking after sample type.

## 4. Discussion

First, using a traditional ANOVA approach, we showed that the samples varied widely in their perceived sensory attributes. Consistent with its description as a high-acid fruit juice [43], CJ (without added sugar) was extremely sour and lacking in sweetness. CJ also exhibited moderately-high bitterness, astringency, cranberry flavor, and overall flavor and was disliked by subjects as a whole. In contrast, CPE was characterized as high in bitterness and astringency, relative to all other samples, but was weak in intensity for all other attributes. Liking for CPE was also low. This was expected since CPE was used purely as a model system to assess the perception of native cranberry polyphenols without the presence of other fruit constituents or added ingredients.

Surprisingly, supplementing the CJC samples with CPE did not alter sourness or cranberry flavor, and astringency perception only increased in CJC with the highest concentration of CPE. However, addition of CPE progressively decreased sweetness, and increased bitterness and overall flavor. Further, overall liking was highest for un-supplemented CJC and lowest for CJC supplemented with the highest concentration of CPE. Notably, un-supplemented CJC was as well liked as CJC supplemented with moderate amounts of CPE (Low and Med samples). These data suggest that adding moderate concentrations of native polyphenols to a formulated cranberry beverage alters its sensory profile but may not have a specific or singular effect on its perceived astringency. Nor would these additions negatively drive liking. These observations may be important for designing acceptable fruit beverages with added polyphenols to enhance flavor and health benefits.

We also found that a moderate level of thickness was perceived in all the fruit-derived samples (except the CPE solution) that is likely associated with the presence of pectin from the fruit [24]. We can only speculate as to why the perception of thickness increased with increasing CPE content of the samples. It is possible that CPE increased the sub-qualities of astringency including roughing, drying, or puckering which was described by Lee & Lawless [1] and reported to occur in other studies on astringency [36,47,48,49]. Subjects in our study may not have associated these sub-qualities with the astringency attribute. Rather, they might have interpreted these mouthfeel cues as texture and ‘dumped’ them in the thickness category. These data suggest that future studies seeking to understand the complexities of astringency perception should conduct a more fine-grained evaluation of this attribute, including its various sub-qualities.

Our prior work [31] showed an interaction between PROP taster status and gender on the perception of bitterness and astringency and overall liking of CJC with added tannic acid (to modify astringency). Based on these earlier results, we expected to find similar robust effects here. However, in the present study we observed no differences in bitterness or astringency of these samples with respect to PROP taster status. ANCOVA did show PROP- specific effects for other attributes such as cranberry flavor (higher intensities experienced by STs) and overall liking (lower in STs). A few PROP taster status by gender interactions were also observed for sourness and cranberry flavor (with female STs giving the highest ratings). However, in general, PROP taster status did not have systematic and wide-spread effects on the perception of our samples.

Previous studies, including our own [31], reported higher perceived bitterness and/or astringency perception by STs (males) in foods supplemented with extraneous materials such as alum, tannic acid, and grapeseed tannins that are not normally used as ingredients in commercial foods. These strategies might have led to an overestimation of the effects of astringent molecules on the perceptions of STs. In the present study, when native cranberry polyphenols were added to cranberry beverages, STs interpreted the samples as having more sourness and flavor but not more bitterness and astringency. One interesting observation from the ANCOVA results was that STs, specifically female STs, perceived more cranberry flavor intensity from the CJC samples relative to their comparison groups. Recent work [50] has shown that astringency associated with polyphenols can be a main modulator of flavor. Thus, it is possible that the addition of CPE did not influence astringency perception, per se, in STs, but these additions were interpreted instead as flavor attributes. These findings suggest that STs may be more sensitive to polyphenol-linked flavor changes in complex foods or beverages. In some respects, the present data evoke the earlier findings reported by Tepper and colleagues (2009) that in comparison with NTs, STs perceived more flavor intensity from commercial samples (i.e., not supplemented) of black coffee and dark chocolate but not necessarily more bitterness from these foods [51]. Thus, a sole focus on perceived differences in bitterness and astringency between NTs and STs may miss important differences in how these two groups appreciate polyphenol-rich foods.

We also found that ethnicity, rarely considered in studies on astringency, was related to the perceptions of bitterness and astringency and to the liking of the CJC samples. While the precise reasons for these differences are unknown, we hypothesize that ethnicity is likely a proxy for exposure to foods that are rich in polyphenols or marked by bitter and astringent sensations. While genetic differences could certainly play a role, the ethnicity-related effects observed here could stem from cultural differences [52,53,54] in familiarity with and acceptance of bitter and astringent fruits and vegetables. Subjects in our study described themselves as either Caucasian or Asian (including East Asian and South Asian). Other researchers have found differences in perception and liking of common odors in similar populations [54,55]. Recent work has shown that these ethnic/cultural differences also extend to taste perception [56], but such studies have been rare. Pedrotti and colleagues (2019) demonstrated that Asian-Chinese subjects perceived greater flavor intensity from mint-flavored chewing gum than Caucasian-European subjects [57]. Further, Yang and colleagues (2020) found associations between ethnicity, sourness perception, and the metallic taste genotype [38]. Specifically, they observed that Asian subjects had higher predicted ratings for both sourness and metallic taste intensity than Caucasian subjects. Thus, evidence is emerging that ethnicity affects the oral experience of sour and astringent foods and beverages and thus needs to be characterized better.

We then used regression trees to visualize the effects of various factors on each of the sensory attributes. In general, the trees and ANCOVA results produced models with similar overall structure. First, sample type was always the first branch to be generated among the trees and thus accounted for the most variation in the dataset. Second, for many of the attributes (although not all of them), CJ and CPE samples were grouped separately from the CJC samples. This was generally consistent with the ANCOVA findings which showed that these two samples were often perceived as outliers and distinct from CJC sample types.

With respect to sourness and cranberry flavor, the regression trees and ANCOVA produced similar results. Gender differences were a distinguishing factor for sourness perception in both models. For cranberry flavor, the PROP taster by gender interaction was a defining feature of the ANCOVA model and this was reflected in separate nodes for PROP and gender in the regression tree for this attribute. However, one cannot conclude that one modeling approach was superior to the other in explaining the data. For example, both ethnicity and gender were factors in the regression tree for bitterness perception, whereas ANCOVA only identified ethnicity as a salient feature of bitterness perception. Conversely, ANCOVA identified age, PROP status and ethnicity as factors for overall liking, but the regression tree only identified ethnicity as a factor for overall liking. Recently, other researchers have utilized regression trees to visualize that sensory perception is multifactorial in nature. Yang and colleagues (2020), demonstrated that PROP taster status, ethnicity and thermal taster status influence sourness and metallic taste perception [38] while Robino et al. (2022) showed that TAS2R38 genotype, PROP phenotype and gender affect basic taste perception, pungency and astringency perception [58]. Thus, regression trees are advantageous in visualizing complex and hierarchical relationships.

However, a well-known concern with regression tree analysis is the risk of overfitting data [46]. As a check on the validity of the regression trees, we used the importance scores generated with random forest modeling (see Table 4) and observed that sample type dominated as the most important factor influencing the sensory attributes and liking. Despite some inconsistencies, the rank order of relative importance scores was generally consistent with the patterns observed in regression trees. For instance, for sourness and cranberry flavor, both PROP taster status and gender were high-ranking features of importance, which agrees with the interactions observed through ANCOVA and the regression trees. They also emphasize our conclusions that PROP status is only one factor among multiple factors contributing to sensory perceptions of foods and beverages. Thus, using the two machine learning approaches and ANCOVA, we arrived at fairly similar conclusions. However, care should be taken against overinterpreting results of these tools. The true value of machine learning approaches is in modeling ‘big’ data. Although the present dataset is relatively small, we showcase a way to use random forest modeling that can be later adapted for larger sensory studies.

Another minor observation in this study was that BMI significantly influenced overall flavor perception. It is widely reported that BMI and taste perception may be related but this is under-studied in complex food models and findings vary among studies [59]. For instance, in another study looking at polyphenol-supplemented wine-like beverage, BMI influenced bitterness and astringency perception [60], but not flavor, opposite to our findings. The reason for the differences among these findings is unclear and additional research is essential to address the role of BMI in flavor perception.

The present study has several strengths. First, we had a robust sample size for a laboratory-based study with approximately balanced PROP taster*gender subgroups. Secondly, we used a food ingredient that is already marketed and accepted by consumers in a commercial beverage (Ocean Spray Inc., Middleborough, MA, USA), and we tested concentrations within the range of sensory palatability. The findings of the present study must also be interpreted in consideration of certain limitations. Our screening procedure only recruited subjects who were familiar with cranberry juice. We did not see major individual differences in liking, which could be a consequence of the nature of the study population i.e., only acceptors of cranberry juice were recruited into the study. In addition, we only examined a limited number of factors in this study, and a wide range of personal traits have been associated with astringency perception including alexithymia, variety seeking, sensitivity to reward and others [22,23]. Future work should incorporate these factors into studies using regression trees and other data analysis approaches to develop more informed models of human responses to astringency.

## 5. Conclusions

The present study reveals some insights as to why research studies on astringency perception often yield inconsistent findings. To be more ecologically relevant, laboratory studies should focus on testing more realistic foods and beverages and supplement them with native polyphenols or polyphenolic compounds that represent everyday foods. Additionally, we showed that various personal factors including PROP status, ethnicity, and gender together may shape the perception and liking of polyphenol-rich foods. These studies demonstrate that it is possible to formulate juice products with a high polyphenol content that can appeal to diverse consumers. These studies should be repeated in a large, representative consumer cohort to determine if the outcomes observed here generalize to consumers-at-large.

## Figures and Tables

**Table 1:** Sample formulations.

Sample	Details
CJ	Cranberry Juice, unsweetened
CPE	0.75 g/L Cranberry Polyphenol Extract in spring water
CJC	Cranberry Juice Cocktail, CJ with 8.75% *w/v* sugar
Low	0.30 g/L CPE in CJC
Med	0.50 g/L CPE in CJC
High	0.75 g/L CPE in CJC

**Table 2:** Subject Characteristics.

Gender	PROP Classification	*n* (of 125)	Ethnicity (*n*)		Age	BMI
			Caucasian	Asian	(Years)	(kg/m^2^)
Female	NT	30	22	8	22.1 ± 1.0	25.2 ± 0.9
	ST	33	23	10	22.2 ± 0.9	24.5 ± 1.0
Male	NT	30	13	17	21.1 ± 0.4	24.8 ± 0.8
	ST	32	19	13	21.7 ± 0.7	25.0 ± 0.6

**Table 3:** ANCOVA model results. Effects of main factors (Sample Type, PROP Taster Status, Gender and Ethnicity) on sensory attribute intensity and overall liking ratings of cranberry juice cocktail samples when adjusted for covariates (Age, BMI). * Only significant interactions are shown ^1,2^.

	Sweetness	Sourness	Bitterness	Astringency	Thickness	Cranberry Flavor	Overall Flavor	Liking
F	**22.402**	**23.778**	**4.274**	**4.494**	**4.419**	**26.048**	**15.699**	**33.643**
Pr > F	**<0.0001**	**<0.0001**	**<0.0001**	**<0.0001**	**<0.0001**	**<0.0001**	**<0.0001**	**<0.0001**
Age	0.116	0.445	1.886	0.769	0.010	0.594	**13.208**	**4.359**
	0.733	0.505	0.170	0.381	0.920	0.441	**0.000**	**0.037**
BMI	3.180	1.217	1.750	0.464	0.391	0.033	**4.316**	2.990
	0.075	0.270	0.186	0.496	0.532	0.856	**0.038**	0.084
PROP Taster Status	0.926	2.947	1.055	0.392	0.001	**5.640**	1.117	**4.546**
	0.336	0.086	0.305	0.531	0.974	**0.018**	0.291	**0.033**
Gender	0.145	2.925	1.593	0.472	2.966	0.118	2.536	1.794
	0.703	0.088	0.207	0.492	0.085	0.731	0.112	0.181
Sample Type	**113.424**	**112.270**	**12.617**	**18.750**	**20.823**	**128.417**	**74.343**	**167.699**
	**<0.0001**	**<0.0001**	**<0.0001**	**<0.0001**	**<0.0001**	**<0.0001**	**<0.0001**	**<0.0001**
Ethnicity	1.118	1.795	**23.297**	**9.239**	0.344	1.189	0.073	**14.136**
	0.291	0.181	**<0.0001**	**0.002**	0.558	0.276	0.787	**0.000**
Taster*Gender	0.413	**12.183**	0.191	1.955	0.463	**14.980**	3.147	0.335
	0.520	**0.001**	0.662	0.162	0.496	**0.000**	0.076	0.563
Gender*Sample	0.486	**2.841**	1.123	0.464	0.228	0.492	0.327	0.728
	0.787	**0.015**	0.346	0.803	0.950	0.782	0.897	0.602

^1^ For each independent variable, the first row shows the *F*-value, while the second row shows the associated *p*-value. ^2^ Values in the table in boldface indicate statistically significant differences (*p* < 0.05).

**Table 4:** Feature importance heatmap based on random forest modeling ^1^.

Features	Sweetness	Sourness	Bitterness	Astringency	Thickness	Cranberry Flavor	Overall Flavor	Liking
Sample	154.39	143.60	28.73	45.69	52.99	145.55	95.62	169.70
Taster	10.60	20.22	0.91	2.55	−0.01	12.26	8.53	3.05
Gender	3.19	19.54	3.11	0.76	−1.67	11.41	7.83	4.80
Ethnicity	4.11	−1.32	15.29	5.60	2.59	4.04	−1.29	8.68
Age	3.10	4.07	4.18	−1.31	6.84	2.07	2.36	3.58
BMI	11.30	6.90	−1.13	1.67	0.19	6.97	16.2	2.74

^1^ Features highlighted in dark green account for the most variation within the data for a particular attribute; a lighter color explains less variation within the data; scores should only be compared down a column and not across rows.

## Data Availability

Data available on request from the authors.
